# Marseillevirus in the Pharynx of a Patient with Neurologic Disorders

**DOI:** 10.3201/eid2211.160189

**Published:** 2016-11

**Authors:** Sarah Aherfi, Philippe Colson, Didier Raoult

**Affiliations:** Aix-Marseille University, Marseille, France; Institut Hospitalo-Universitaire (IHU) Méditerranée Infection, Marseille

**Keywords:** Marseillevirus, viruses, giant virus, Megavirales, megavirome, neurologic disorders

**To the Editor:** *Marseilleviridae* is a recently described family of giant amebal viruses (*1*). Although Marseillevirus, its founding member, and subsequently discovered representatives were isolated primarily from environmental water, marseilleviruses have been recovered from humans (*2**,**3*). Senegalvirus, a close Marseillevirus relative, was serendipitously isolated from a healthy man’s feces (*2*). Metagenomics then unexpectedly identified Marseillevirus-related sequences in blood of healthy donors (*3*), which was confirmed by PCR, fluorescence in situ hybridization, and serologic testing. Further PCR and serologic studies suggested substantial exposure of humans to marseilleviruses (*4**,**5*).

During assessment of Marseillevirus serology at Institut Hospitalo-Universitaire (IHU) Méditerranée Infection (Marseille, France), we found serum from an 11-month-old boy with lymphadenitis that exhibited a high Marseillevirus IgG titer; the virus was detected by PCR in serum and by fluorescence in situ hybridization and immunohistochemistry in the lymph node (*6*). Subsequently, the hospital implemented systematic Marseillevirus PCR in cases of gastroenteritis or pharyngitis, which led to detection of Marseillevirus DNA in pharyngeal and blood samples from a 20-year-old man. He had sought treatment in November 2013 for a 2-day febrile gastroenteritis that was treated with amoxicillin and acetaminophen; however, several hours later, his fever reached 40°C, and intense headache and stiff neck led to his hospitalization. No adenopathy was palpable. Laboratory analyses showed elevated C-reactive protein (194 mg/L), elevated bilirubin (44 µmol/L), low platelet count (120 G/L), and elevated polynuclear cell count (9 G/L). Cerebrospinal fluid (CSF) was clear and acellular; the CSF to blood glucose ratio was normal, but the protein level was elevated (0.73 g/L).

We tested CSF and feces by culture, PCR, or immunoenzyme assay for common infectious agents of meningitis, encephalitis, and gastroenteritis, including enteroviruses, herpesviruses, *Neisseria meningitidis*, *Streptococcus pneumoniae*, caliciviruses, rotavirus, adenoviruses, and *Clostridium difficile*. All results were negative. Feces were also negative for Marseillevirus DNA. Serologic test results were negative for HIV and cytomegalovirus. However, a pharyngeal sample was positive for Marseillevirus in routine diagnosis using the PCR system ORF152 (*3*); sequencing showed 100% nucleotide identity with the Marseillevirus genome (http://www.mediterranee-infection.com/article.php?laref=495&titre=marseillevirus-pharynx). Retrospective testing of CSF for Marseillevirus DNA yielded negative results. 

The patient recovered after receiving ciprofloxacin and was discharged after 72 hours. One year later, he exhibited vertigo and a 7-kg weight loss over 4 months, although no additional episode of gastroenteritis or fever had occurred. He reported a slight impairment of cognitive functions (i.e., memory, attention), but clinical examination and cerebral positron emission and computed tomographic scan results were normal. Vertigo was attributed to vestibular deficiency and treated with betahistine. CSF testing still showed an isolated high protein level (0.68 G/L) without hypercellularity but negative results for bacteria and viruses. However, Marseillevirus DNA was detected by 2 PCR systems that target a helicase gene: blood testing using the ORF152 PCR and pharyngeal swab specimen testing using the HelF6R6 PCR system (primers: 5′-GAGGATGTACGGAAGGTC-3′ [forward]; 5′-GTCCTTCACCTGTTCTTCC-3′ [reverse]). Sequence identities were 99% and 100%, respectively, with Marseillevirus (GenBank accession no. KU933837; http://www.mediterranee-infection.com/article.php?laref=495&titre=marseillevirus-pharynx). In addition, Marseillevirus IgG was detected by indirect immunofluorescence assay; serum samples that were negative or positive for Marseillevirus IgG in previous experiments were used as negative and positive controls, respectively (*4*). After 2 months, the patient’s general condition had improved, and neurocognitive and vestibular symptoms resolved.

Marseillevirus presence in the case-patient is indisputable, as supported by specific molecular detection and sequencing of 2 sequential pharyngeal swab specimens and of blood, with concurrent IgG positivity (Figure). The presence of the virus in 2 samples collected at a 1-year interval suggests chronic carriage. 

**Figure:**
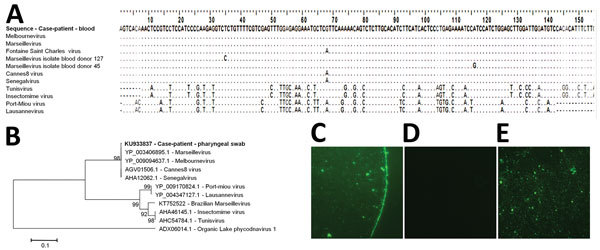
Marseillevirus sequences and serologic analysis for a 20-year-old man in Marseille, France, who initially sought treatment in November 2013 for a 2-day febrile gastroenteritis. A) Alignment of the sequence obtained in November 2014 from the blood of the case-patient with sequences from Marseillevirus and other related viruses. GenBank accession nos.: Marseillevirus, GU071086.1; Melbournevirus, KM275475.1; Fontaine Saint-Charles virus, KF582416.1; Senegalvirus, KF582412.1; Marseillevirus isolate blood donor 127, KF233993.1; Marseillevirus isolate blood donor 45, KF233992.1; Cannes 8 virus, KF261120.1; Tunisvirus, KF483846.1; Insectomime virus, KF527888.1; Port-Miou virus, KT428292.1; Lausannevirus, HQ113105.1. B) Phylogenetic reconstruction based on an amino acid alignment of the translated sequence obtained in November 2014 from a pharyngeal swab specimen from the case-patient (GenBank accession no. KU933837; indicated in bold) and homologous sequences from Marseilleviruses. Sequence from Organic Lake phycodnavirus 1 was used as an outgroup. The evolutionary history was inferred in MEGA6 software (http://www.megasoftware.net/) by the neighbor-joining method. The percentage of replicate trees in which the associated taxa clustered together in the bootstrap test (1,000 replicates) is shown next to the branches. The evolutionary distances were computed by using the Kimura 2-parameter method. The tree is drawn to scale, with branch lengths in the same units as those of the evolutionary distances used to infer the phylogenetic tree. Scale bar indicates nucleotide substitutions per site. C–E) Marseillevirus IgG detection by immunofluorescence in a serum sample from the case-patient. C) Serum sample from the case-patient at dilution 1:50; D) negative control (serum sample from a rabbit not exposed to Marseillevirus) at dilution 1:50; E) positive control (serum sample from a rabbit exposed to Marseillevirus) at dilution 1:50.

Several reports showed that giant viruses may be common in humans, but association with pathogenicity was documented differently, depending on the viruses. Thus, many serologic, virologic, and clinical findings argued for a causative role of mimiviruses in pneumonia, which was strengthened in 2013 by the culture isolation of mimiviruses from 2 pneumonia patients (*7**,**8*). In addition, *Acanthocystis turfacea* chlorella virus-1, a phycodnavirus that infects algae, was detected by metagenomics in human oropharyngeal samples, and this association was further confirmed by PCR in 92 samples, with a prevalence of 44% (*9*). Unexpectedly, DNA detection of this virus was associated with a decrease in cognitive performance in these patients; such cognitive disorders were also observed in mice inoculated with this virus.

The presence of Marseillevirus in healthy humans was described by high-throughput sequencing and subsequent culture isolation from feces (*2*), then by metagenomics in blood donors’ blood (*3*). Unexpectedly, seroprevalence studies conducted in the general population showed high (up to 13%) positivity rates of Marseillevirus IgG, which suggested a common human exposure (*3**–**5*). Presence of Marseillevirus in a symptomatic human was reported in 2013 in an 11-month-old boy with lymphadenitis and possibly corresponded to a primary infection (*6*). Marseillevirus was then detected in the lymph node of a 30-year-old woman with Hodgkin’s lymphoma (*10*). In the case we describe, Marseillevirus was detected in the human oropharynx in association with cognitive impairment and possible chronic carriage with concurrent persistence of clinical signs. The involvement of Marseillevirus in these symptoms cannot be established here, but these findings warrant further investigation.
